# Near-Wall Slow Flow Contributes to Wall Enhancement of Middle Cerebral Artery Bifurcation Aneurysms on Vessel Wall MRI

**DOI:** 10.3390/diagnostics14232722

**Published:** 2024-12-03

**Authors:** Linfeng Liu, Zhuhao Li, Weiping Xiao, Yibing Yang, Yan Yan, Lu Bai, Lingzhi Quan, Tiewei Qi, Feng Liang

**Affiliations:** 1 Department of Neurosurgery, Sun Yat-sen University First Affiliated Hospital, Guangzhou 510060, China; 2 Department of Radiology, Sun Yat-sen University First Affiliated Hospital, Guangzhou 510060, China

**Keywords:** middle cerebral artery bifurcation aneurysms, aneurysm wall enhancement, silicone models, aspect ratio, near-wall slow flow

## Abstract

**Background:** The mechanism of aneurysm wall enhancement (AWE) in middle cerebral artery (MCA) bifurcation aneurysms on vessel wall magnetic resonance imaging (VW-MRI) remains unclear. We aimed to explore the morphologically related hemodynamic mechanism for the AWE of MCA bifurcation aneurysms. **Methods:** Patients with unruptured MCA bifurcation aneurysms undergoing VW-MRI were enrolled. Logistic regression analyses were performed to determine the risk factors for AWE. Based on the results of retrospective analyses, bifurcation aneurysm silicone models with a specific aspect ratio (AR) were designed and underwent VW-MRI with different inlet velocities. Computational fluid dynamics (CFD) analyses were conducted on both silicone models and patients’ aneurysms. **Results:** A total of 104 aneurysms in 95 patients (mean age 60; 34 males) were included for baseline analysis and morphological analysis. Logistic regression analysis indicated AR (OR, 5.92; 95% CI, 2.00–17.55; *p* = 0.001) was associated with AWE. In the high-AR group of 45 aneurysms with AWE, the aneurysm sac exhibited lower blood flow velocity, lower wall shear stress, a larger proportion of low-flow regions and higher wall enhancement values. In total, 15 silicone models were analyzed, divided into three subgroups based on neck width (4 mm, 6 mm, and 8 mm). Each subgroup contained aneurysms with five different ARs: 1.0, 1.25, 1.5, 1.75, and 2.0. In silicone models, contrast enhancement (CE) was mainly located beneath the dome of the aneurysm wall. With the same inlet velocity, CE gradually increased as the AR increased. Similarly, at the same AR, CE increased as the inlet velocity decreased. CFD demonstrated a moderate positive correlation between the near-wall enhancement index and the ratio of the low-velocity area (r = 0.6672, *p* < 0.001). **Conclusions:** The AR is associated with the AWE of MCA bifurcation aneurysms. A high AR may promote wall enhancement by causing near-wall slow flow.

## 1. Introduction

Middle cerebral artery (MCA) aneurysms, mainly on the bifurcation, account for a third of intracranial aneurysms [1]. Both endovascular treatment and surgical clipping could secure unruptured MCA aneurysms, but they also carry the risk of complications [2,3,4]. Therefore, it is highly desirable to identify unstable unruptured MCA aneurysms when making clinical decisions.

Aneurysmal wall enhancement (AWE) on vessel wall magnetic resonance imaging (VW-MRI) can provide valuable information for the risk stratification of aneurysms [5,6]. It is suggested that AWE is more likely to be observed in aneurysms with a larger size, a larger aspect ratio (AR), irregular regions and daughter sacs [6,7]. But the role of parameters in causing AWE and how they influence the enhancement patterns remain unclear.

Previous studies have shown that AWE is associated with inflammatory cell invasion, neovascularization and atherosclerotic changes [8,9,10]. However, in samples of aneurysms without AWE, the presence of related inflammatory substances can also be observed [11]. These results suggest that inflammation is not the sole factor contributing to AWE, and the presence of inflammation does not necessarily indicate that an aneurysm will develop AWE.

Some studies have demonstrated correlations between AWE and morphological parameters as well as the hemodynamics of aneurysms [7,12,13]. Aneurysm morphology is associated with blood flow patterns within the aneurysm sac [14,15]. The altered flow conditions in aneurysms can further impact biological processes of vessels [16].

However, few previous studies have systematically and quantitatively investigated the impact of changes in intraluminal blood flow patterns on the distribution of contrast agents retained within the MCA bifurcation aneurysm and further explored the relationship between aneurysm morphological parameters and near-wall slow flow. In this study, we analyzed MCA bifurcation aneurysms and artificially designed bifurcation aneurysm silicone models, utilizing computational fluid dynamics (CFD) and in vitro MRI examination in order to explore the morphologically related hemodynamic mechanism for AWE.

## 2. Materials and Methods

### 2.1. Patient Selection

**Figure 1:**
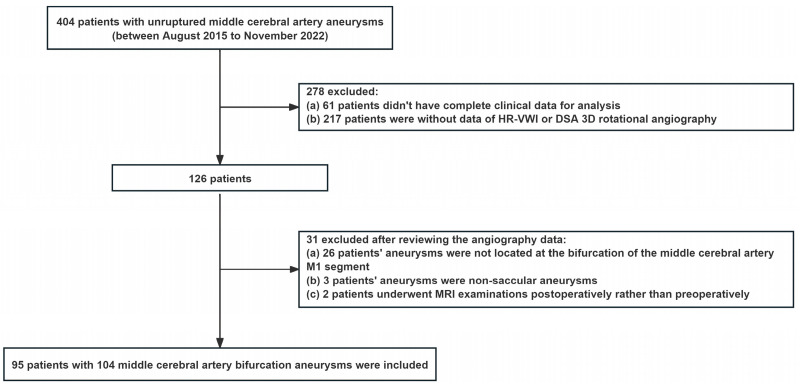
Patient flowchart of the study.

Consecutive patients with the diagnosis of MCA bifurcation aneurysms from August 2015 to November 2022 at our institution were retrospectively analyzed (Figure 1). The inclusion criteria were as follows: (1) diagnosed as unruptured aneurysms located at the bifurcation of the MCA M1 segment by at least 2 neurosurgeons with 5 years of clinical experience or above; (2) available original data of VW-MRI and DSA 3D rotational angiography; and (3) complete clinical data for analysis. The exclusion criteria were as follows: (1) non-saccular aneurysms like fusiform or dissecting aneurysms; (2) previously clipped or coiled aneurysms; (3) VW-MRI or DSA 3D rotational angiography images with poor imaging quality that could not be analyzed; and (4) patients with vascular diseases that may affect the hemodynamic analysis of the aneurysm, such as vascular malformations, severe middle cerebral artery stenosis or occlusion.

### 2.2. VW-MRI Protocol and Definition of AWE

VW-MRI was performed in a 3.0 T clinical MR imaging scanner Magnetom Verio (Siemens, Erlangen, Germany) with a 12-channel head coil. The main scan sequences included 3D TOF MRA, turbo spin echo (TSE) T1WI plain and enhanced scanning, and TSE T2WI plain scanning. Meanwhile, 3D T1WI plain and enhanced scanning were added. Gadopentetic acid (Gd-DTPA) was used as the contrast agent.

The T1WI scanning parameters were as follows: repetition time (TR) = 650 ms, echo time (TE) = 12 ms, field of view (FOV) = 130 × 130 mm, matrix 256 × 256, slice thickness 1.5 mm, slice gap 0.15 mm, flip angle 145°, voxel size 0.5 × 0.5 × 1.5 mm, and scanning time 4 min 40 s. The scanning scope typically ranged from 9 to 13 slices, depending on the size of the aneurysms. Axial scanning was performed in all patients.

The 3D T1WI parameters were as follow: TR = 800 ms, TE = 20 ms, FOV = 250 × 206 mm, matrix 320 × 264, slice thickness 0.78 mm, slice gap 0 mm, voxel size 0.78 × 0.78 × 0.78 mm, and mean scanning time 6 min 51 s [17].

The signal intensity of the aneurysmal wall on VW-MRI-enhanced T1WI was adjusted by that of the pituitary stalk to calculate a contrast ratio (CR_stalk_), and CR_stalk_ ≥ 0.60 was considered an enhancement [18].

### 2.3. Measurement for Morphology Parameters

Moreover, 3D models were reconstructed using the patients’ 3D rotational angiography data in Mimics 21.0 (Materialise, Leuven, Belgium) by L.L., who holds a certification in biofluid mechanics modeling and simulation engineering. The same threshold parameters were used for vascular structure segmentation in all cases to ensure consistency. Morphological parameter measurement was conducted in 3-matic 21.0 (Materialise, Leuven, Belgium) (Figure S1). All measurements were carried out by Z.L and W.X. with over 5 years of experience in interpreting neuroimaging, and then the average value was used. For discrepancies greater than 10%, the results were discussed and remeasured. These parameters were defined and measured as follows:

1. Height: the maximal diameter from the centroid of the neck plane to the aneurysm dome.
2. D_max_: the largest measurement in terms of the maximum dome diameter or width.
3. Neck width: the width of the neck plane (the junction between the aneurysm and the parent artery, specifically the base of the aneurysmal protrusion).
4. AR: the ratio of height/neck width.
5. Average diameter of parent artery: (D1a + D1b + D2a + D2b + D3a + D3b)/6
6. Size ratio: the ratio of height/average diameter of the parent artery.
7. Surface area (S): the surface area of the actual aneurysm that grew from the junction of the aneurysm with its parent artery.
8. Volume (V): the volume of the actual aneurysm that grew from the junction of the aneurysm with its parent artery.
9. Nonsphericity index: calculated using the formula [19]: $1-(18\pi {)}^{\frac{1}{3}}\times \frac{V^{\frac{2}{3}}}{S}$.

### 2.4. Design of Bifurcation Aneurysm Silicone Models

**Figure 2:**
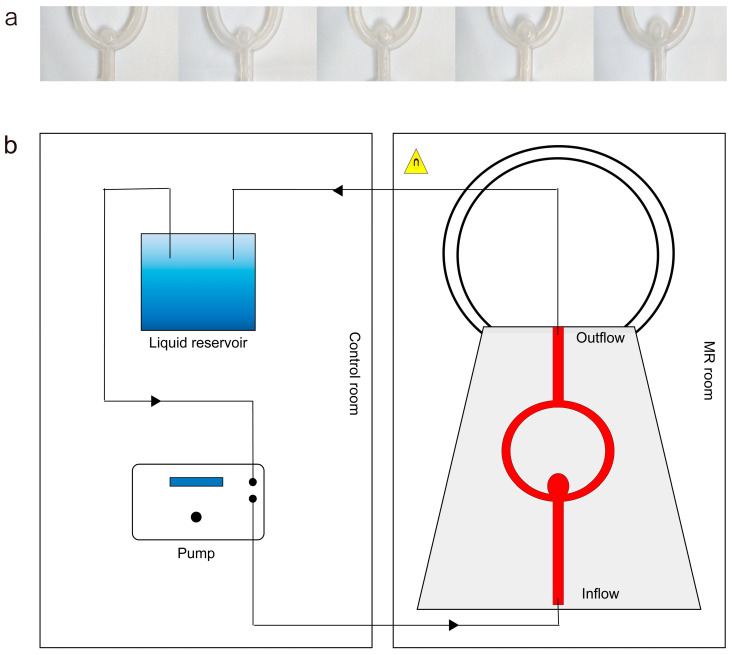
(**a**) Aneurysm silicone models with the same neck width but different AR (from left to right, AR = 1.0, 1.25, 1.5, 1.75, 2.0). (**b**) Piping system of the silicone models to MR examination.

In order to study the impact of varying flow and morphological parameters on AWE, we decided to create in vitro silicone models of bifurcation aneurysms based on the results of retrospective analyses. Aneurysm molds and models were designed using Geomagic Design Direct 2014 (3D Systems, Rock Hill, SC, USA). Highly curved vessels and unnecessary branches can complicate flow fields, increasing flow instability and vortex formation. A straight artery provides more stable and predictable flow conditions, enhancing model reproducibility. Additionally, the aneurysms studied were located at the M1 bifurcation, where the M1 segment was also relatively straight. Hence, the models were designed (Figure 2a). The diameter of the parent vessel lumen was 4 mm, and the average wall thickness of the silicone models was approximately 1.8 mm. Aneurysm silicone models were divided into 3 subgroups with neck width of 4 mm, 6 mm, and 8 mm. Each subgroup contained 5 aneurysms with ARs of 1.0, 1.25, 1.5, 1.75, and 2.0. Then, the molds were printed and produced by the silicone 3D printer Lite 600 (UnionTech, Shanghai, China) using photosensitive resin. The surface of the molds was polished and smoothed to ensure the surface finish of the vessels. The model material was made of high-transparent silica gel (Dongguan Guochuang Silicone Material Co., Ltd., Dongguan, Guangdong, China) with a Shore hardness of 30 A.

### 2.5. MRI Protocol for Silicone Models

The silicone model was immersed in the gel (GUANGGONG, Guangzhou, China), with its inlet connected to a pump (YANXIAO, Shanghai, China) (Figure 2b). With the glycerol–sterile saline mixture (40:60) being injected into the model at varied velocities (0.1 m/s, 0.2 m/s, 0.3 m/s, 0.4 m/s, 0.5 m/s, and 0.6 m/s), T1WI scanning was conducted. Then, a concentration of 0.5 mM Gd-DTPA dissolved in saline mixture was injected into the models at the same velocities and enhanced T1WI was performed [20]. The whole model system would be washed thoroughly between trials.

MR was performed in the 3.0 T clinical MR scanner Magnetom Verio (Siemens, Erlangen, Germany) with an 8-channel knee coil. Pre- and post-contrast motion-sensitized driven equilibrium (MSDE)-prepared 3D T1 sampling perfection with application-optimized contrasts by using different flip angle evolutions (3D T1-SPACE) was used. The SPACE parameters were as follow: TR/TE = 800/16 ms; slice thickness = 2 mm; FOV = 154 × 154 mm; matrix = 256 × 256; voxel size = 0.6 mm; MSDE preparation mode: first gradient moment 300 mT × ms^2^/m; and scanning time 1 min 48 s [21].

### 2.6. Radiological Analysis of Models

In order to assess the near-wall enhancement strength within the aneurysmal sac, we established a parameter called the near-wall enhancement index (*NWEI*), which was calculated as the formula below [22]:

$$NWEI=\frac{\frac{SI\_{Nearwall}_{postcontrast}}{{SI\_Background}_{postcontrast}}-\frac{{SI\_Nearwall}_{precontrast}}{{SI\_Background}_{precontrast}}}{\frac{{SI\_Nearwall}_{precontrast}}{{SI\_Background}_{precontrast}}}$$

*SI* indicates signal intensity.

### 2.7. CFD of Silicone and Patients’ Aneurysm Models

For silicone aneurysm models designed in Geomagic software 2014, STEP files were imported to Fluent Software 19.2 (ANSYS Corporation, Canonsburg, PA, USA). For patients’ aneurysm models, after reconstruction in Mimics21.0 (Materialise, Leuven, Belgium), they were imported into 3-matic 21.0 (Materialise, Leuven, Belgium) for smoothing and surface construction. Then, stereolithography files were imported to Fluent Software 19.2. For meshing, over 4 million polyhedral elements were created, with a maximum mesh size of 0.1 mm, along with 5 layers of wall prism elements to achieve precise boundary layer resolution. All the mesh had a minimum orthogonal quality of over 0.70 and a maximum skewness of lower than 0.5. After meshing, the inlets, outlets, and walls of the aneurysm models were selected. Then, CFD was performed. In brief, the vessel wall was defined as a rigid wall with no-slip boundary conditions, under a steady status for silicone models and under a transient status for patients’ models, respectively. Neglecting the gravity effect, laminar and incompressible flow with density and viscosity values of 1060 kg/m^3^ and 0.004 Pa∙s was imposed on the inlets of the models. Other parameter settings can be found in Table S1. For patients’ aneurysms, the AWE area was detected and reconstructed by iPlan cranial 3.0 (BrainLab, Munich, Germany). Further details of the method can be found in our previous study [13]. We performed hemodynamic parameter analysis using CFD-POST 19.2 (ANSYS Corporation, Canonsburg, PA, USA). The volume-averaged velocity and WSS in the aneurysm dome and neck regions were measured, and their ratios were calculated to normalize the parameters for each aneurysm, ensuring data comparability.

### 2.8. Statistical Analysis

We performed statistical analysis using the statistical software package Stata 17.0 (StataCorp, College Station, TX, USA). Normally distributed data were reported as means with standard deviation (mean ± SD), whereas non-normally distributed data were presented as medians (interquartile ranges). Differences in continuous variables were compared using Student’s *t* tests or Wilcoxon’s rank-sum test. Differences in categorical variables were compared using the chi-square test or Fisher exact test. The statistically significant risk factors (*p* < 0.2) in the univariate analysis were selected for multivariate logistic regression analysis. Stepwise logistic regression models were built to explore the odds and correlates of AWE. A *p* value ≤ 0.05 was considered statistically significant.

## 3. Results

### 3.1. Patient and Aneurysm Characteristics

Ninety-five patients (sixty-one females; age range 41–89 years) with 104 MCA bifurcation aneurysms were included in this study. Nine (9.47%) patients had bilateral MCA bifurcation aneurysms. Based on the CR_stalk_, patients and aneurysms were divided into an AWE group and non-AWE group. All the clinical characteristics of the patients including medical history (hypertension, diabetes, hyperlipidemia, stroke and subarachnoid hemorrhage history), risk and safety factors (currently smoking, alcohol abuse and a history of antiplatelet or lipid-lowering medication use) were not associated with AWE (*p* > 0.05) (Table 1).

### 3.2. AR Is Associated with AWE

The detailed characteristics of the aneurysms with and without AWE are shown in Table 2. AWE could be detected in 46.15% (48/104) unruptured MCA bifurcation aneurysms. Height, D_max_, neck width, the AR, the size ratio, and the nonsphericity index were also associated with AWE (*p* < 0.05). Stepwise logistic regression analysis indicated that the AR (OR, 5.92; 95% CI, 2.00–17.55; *p* = 0.001) was associated with AWE.

### 3.3. Low Near-Wall Velocity Induced by High AR Contributes to AWE

The median AR value was 1.62, which was close to the cut-off value associated with ruptured aneurysms [23,24]. In total, 48 patients’ aneurysms with AWE were divided into a low- and a high-AR group according to the median AR value (Table 3). In the high-AR group, the aneurysm sac also exhibited lower blood flow velocity [0.0083 m/s (0.0012–0.0161) vs. 0.0354 m/s (0.0225–0.0476), *p* < 0.001] and lower WSS [0.0714 Pa (0.0270–0.2036) vs. 0.6432 Pa (0.4232–1.128), *p* < 0.001]. The high-AR group had lower velocity beneath the enhanced area of aneurysms and lower WSS at the enhanced area of aneurysms normalized by the velocity and WSS of aneurysm necks.

**Figure 3:**
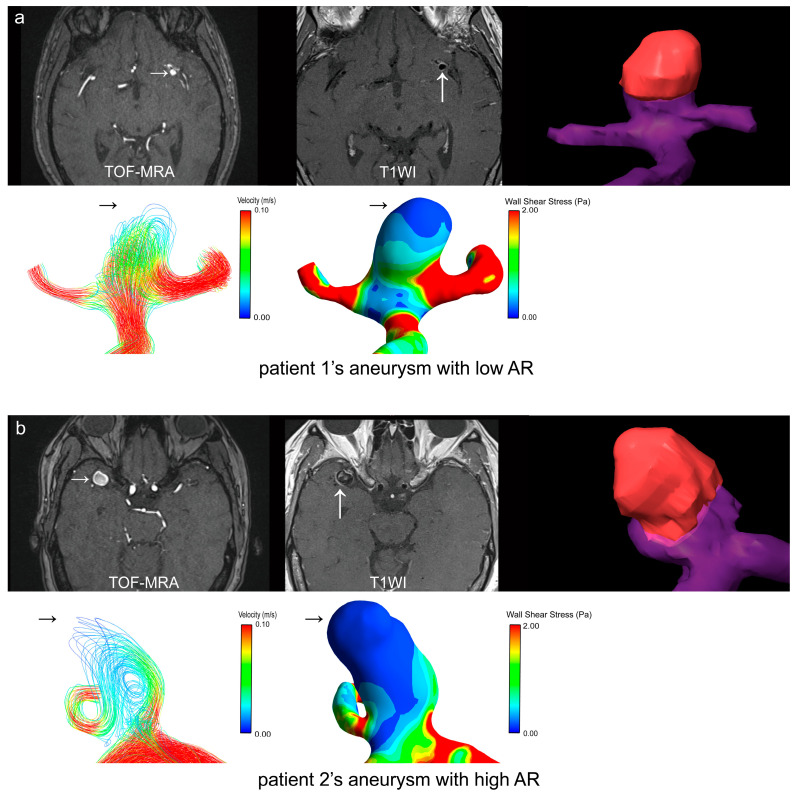
Images of the patients’ aneurysms with different ARs. The auto fusion of the TOF-MRA (small white arrow) and VW-MRI T1WI (large white arrow). The AWE area is highlighted in red and the aneurysm with parent artery is highlighted in purple. (**a**) The enhanced area (black arrow) of the aneurysm with the low AR of patient 1 has a lower proportion of low-WSS and low-velocity regions. (**b**) The enhanced area (black arrow) of the aneurysm with the high AR of patient 2 has a larger proportion of low-WSS and low-velocity regions.

On the other hand, we defined the area with a velocity < 0.004 m/s in patients’ aneurysms as the low-velocity area, then a longitudinal–sectional plane across the center of the aneurysmal enhanced area was made to calculate the low-velocity area ratio (LVAR). Compared with the low-AR group, a larger LVAR was found in the high-AR group [0.4669 (0.2266–0.5601) vs. 0.1209 (0.0932–0.3188), *p* = 0.007] (Table 4), which was moderately correlated with the AR of patients’ aneurysms (r = 0.7087, *p* < 0.001) (Figure S2). Meanwhile, the CR_stalk_ value of the high-AR group was also higher than that of the low-AR group [0.8481 (0.7287–0.9969) vs. 0.7338 (0.6406–0.8476), *p* = 0.037] (Table 4). As illustrated in the examples shown in Figure 3, CFD also confirmed that the enhanced area of the aneurysm with the high AR of patient 2 had larger proportions of low-WSS and low-velocity regions than that in the low-AR aneurysm of patient 1.

**Figure 4:**
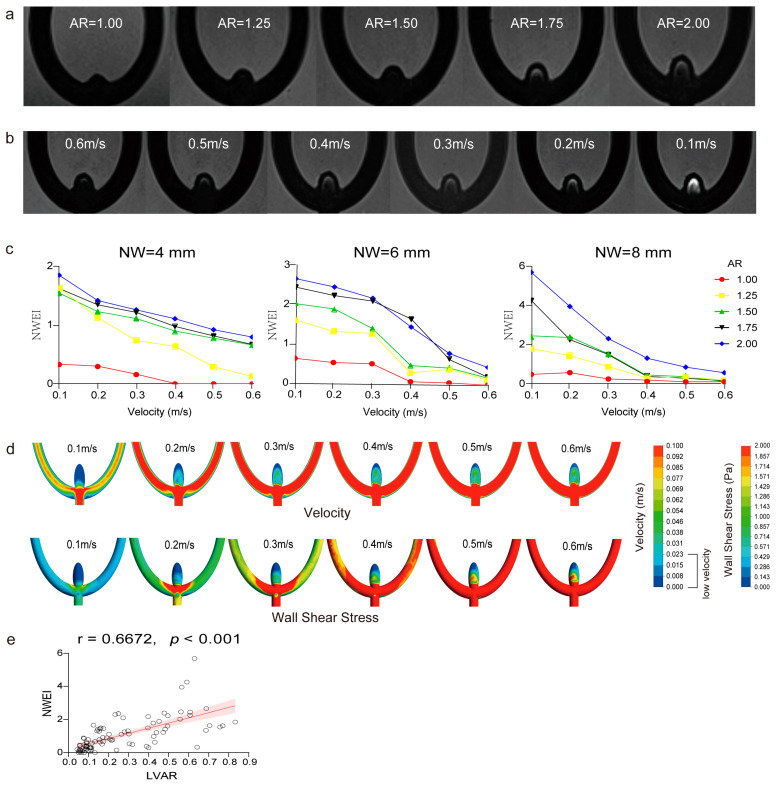
(**a**) Representative pro-contrast images of the silicone bifurcation aneurysms with the same neck width (4 mm) and inflow velocity (velocity, 0.4 m/s), indicating a different AR. (**b**) In the same aneurysm model (AR = 2, neck width = 4 mm), the pro-contrast image shows contrast agent retention with different inflow velocities. As the flow rate decreases, more retention is observed. (**c**) The impact of the variation in inlet flow velocity on NWEI. (**d**) The velocity map of the aneurysm dome section indicates the presence of a low-flow velocity field near the aneurysm dome (AR = 2, neck width = 4 mm), which is related to the inlet velocity. As the inlet velocity decreases, the area of low-flow velocity regions increases. In the same models, the low-WSS regions are enlarged as the inlet velocity decreases. (**e**) A quantitative correlation of the NWEI with the LVAR of the aneurysm silicone models.

To confirm the role of low velocity and WSS in the hemodynamic mechanism of enhancement identified in MR imaging, in total, 15 silicone aneurysm models (neck width = 4 mm, 6 mm, and 8 mm, with an AR = 1.00, 1.25, 1.50, 1.75, and 2.00) were scanned for NWEI quantification. Overall, an elevated tendency of the NWEI was observed when the AR increased in aneurysm models with a certain neck width under a fixed inflow velocity, and the enhancement was mainly located beneath the dome of the aneurysm models. In addition, we found that all the aneurysm models had the largest NWEI under the inflow velocity of 0.1 m/s. When the inflow velocity increased, the NWEI showed varying degrees of decline and fell to the lowest level under the inflow velocity of 0.6 m/s (Figure 4a–c). Then, CFD for the silicone models was performed. Longitudinal–sectional planes correspondingly showed a relatively low-flow velocity field (velocity < 0.023 m/s) beneath the dome of the aneurysm models, accompanied by a low-WSS area around the dome (Figure 4d). A correlation analysis further suggested a moderately positive correlation of the NWEI with LVAR (r = 0.6672, *p* < 0.001) (Figure 4e).

**Figure 5:**
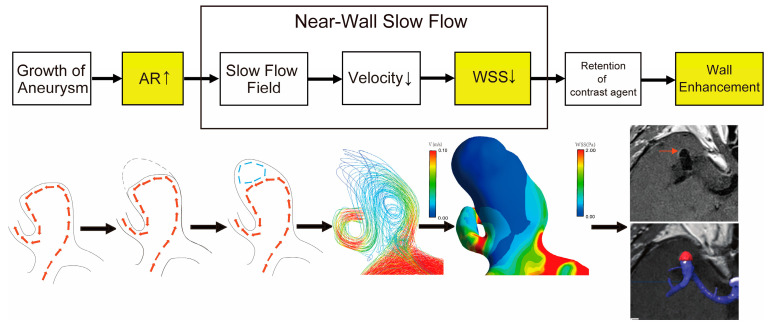
Schematic diagram of the hypothesis of aneurysm wall enhancement. Thick red arrows represent a hypothetical blood flow path, blue arrows indicate low-flow regions, and thin red arrows point to the wall enhancement area (highlighted in red) of the bifurcation aneurysm (highlighted in blue). The upward and downward arrows in the figure indicate the increase and decrease of the relevant variables, respectively.

The above data suggested that a high AR could contribute to the formation of AWE, probably by inducing a low-flow velocity field (Figure 5).

## 4. Discussion

Previous studies on the hemodynamic analysis and AWE of MCA bifurcation aneurysms are limited. In this study, we retrospectively analyzed 104 MCA bifurcation aneurysms from 95 patients, which is a larger sample size than previous reports [8,25,26], demonstrating that the AR was associated with AWE.

Moreover, we found that an enhancement on VW-MRI, potentially due to near-wall contrast agent stasis, could be triggered merely by modifying the inflow velocity or adjusting the aneurysm AR. As the inflow velocity decreased or the AR increased, the contrast enhancement gradually intensified. Therefore, we believe that the potential mechanism of AWE may be that the increasing AR during aneurysm development leads to a decrease in blood flow velocity within the aneurysm sac. This, in turn, creates a low-velocity flow field and low WSS, eventually causing the retention of the contrast agent and an enhancement of the aneurysm wall (Figure 5).

Some studies suggest that the near-wall enhancement (NWE) resulting from slow blood flow may only mimic true wall enhancement and is thus referred to as pseudo-enhancement [27,28]. These aneurysms, which were misjudged as having AWE, may have not yet experienced wall damage or weakening and may be incorrectly classified as high-risk ruptured aneurysms, leading to excessive interventions. However, we demonstrated that a higher AR was associated with slower intracavitary blood flow velocity, lower WSS, larger regions of low flow velocity, and higher CR_stalk_ in MCA bifurcation aneurysms. Meanwhile, in the silicone model experiment of this study, there was no obvious contrast agent retention in the lumen of the round-like low-AR aneurysm with a low rupture risk until the AR was higher than 1.5. Furthermore, NWE induced by slow blood flow tends to occur more frequently in large-volume aneurysms and aneurysms with daughter sacs, considering that the flow in the distal or daughter sacs is slower. These kinds of aneurysms associated with slow blood flow have been proven to be related to AWE and rupture [22,29]. Khan et al. [30] also discovered lower WSS and sac-averaged velocity and a larger aneurysm size in the AWE group than those in non-AWE group. Since the morphological and hemodynamic risk factors for NWE and AWE are consistent, such as a high AR and low WSS or velocity, we believe that the enhancement observed on VW-MRI, whether it is NWE or true AWE, equally indicates a high risk of aneurysm growth and rupture.

In addition, it has been mentioned that slow blood flow with low WSS can induce endothelial deactivation and dysfunction by causing oxidative stress and inflammatory response, leading to vascular degeneration [16,31,32]. Therefore, AWE could be possibly caused by contrast agent uptake or leakage in the slow flow area into a damaged endothelial cell layer due to a congenitally weak focal vessel wall or hemodynamic factor-induced vascular inflammation [33]. The longer the vascular wall remains in a low-flow state, the more damaged the endothelial cells become, and the higher the permeability. The accumulated contrast agent has a greater chance of permeating or adhering to the aneurysm wall, resulting in a higher AWE value. In summary, the presence of NWE suggests the formation of an intraluminal low-flow field, possibly placing the aneurysm wall in a vulnerable environment, making it more prone to AWE.

The increased AR of the aneurysm silicone models could be regarded as a representation of aneurysm enlargement. During this process, the intraluminal blood flow in the aneurysm became slower and more complex, leading to larger enhanced regions with higher enhancement values. And larger and elongated aneurysms have been proven to be more prone to rupture [15]. This could potentially explain the theoretical association of higher wall enhancement values with an increased risk of aneurysm growth or rupture reported in the literature [34].

This study had some limitations. First, this was a single-center study, and we only conducted an analysis of MCA bifurcation aneurysms, and so cautions should be exercised when extrapolating the conclusions to sidewall aneurysms located in sidewall of the artery or other locations. Second, we were unable to obtain longitudinal data on aneurysm enlargement to validate the hypothesis that pseudo-enhancement could transform into true wall enhancement in the future. A larger sample size and sufficient follow-up data are necessary to further investigate this hypothesis through a well-designed research protocol. Third, the differences between the results of CFD analysis and the actual hemodynamic conditions of the aneurysm can be attributed to several factors, including the standardization of inflow conditions rather than personalized ones, assuming blood as a Newtonian fluid, arterial walls as rigid walls, artificial segmentation and reconstruction processes and so on. Fourth, as there were correlations between aneurysmal morphological parameters, during changes in AR, height, neck width, volume, and other physical characteristics of the model also changed accordingly, making it impossible to achieve variable restrictions. Last but not least, the utilization of silicone models had the potential to offer valuable insights into the dynamics of fluid within aneurysms. Nevertheless, the inherent properties of silicone, such as not being tissue-like the aneurysm wall, limited the further elucidation of various crucial aspects.

## 5. Conclusions

The AR is independently associated with AWE in MCA bifurcation aneurysms. A high AR may contribute to the formation of AWE by inducing a low-flow velocity field.

## Figures and Tables

**Table 1:** Clinical characteristics of the patients with and without AWE.

	Non-AWE (*N* = 50)	AWE (*N* = 45)	*p*-Value
Female	34 (68.00%)	27 (60.00%)	0.52
Age (year), mean ± SD	59.68 ± 8.14	60.60 ± 9.46	0.61
Hypertension	39 (78.00%)	30 (66.67%)	0.25
Diabetes	10 (20.00%)	7 (15.56%)	0.60
Hyperlipidemia	21 (42.00%)	14 (31.11%)	0.29
Current smoking	4 (8.00%)	7 (15.56%)	0.34
Alcohol abuse	4 (8.00%)	5 (11.11%)	0.73
History of stroke	14 (28.00%)	7 (15.56%)	0.22
History of subarachnoid hemorrhage	5 (10.00%)	1 (2.22%)	0.21
Using lipid-lowering drugs and anti-platelet drugs for no less than 3 months	11 (22.00%)	8 (17.78%)	0.80

SD, standard deviation.

**Table 2:** Characteristics of aneurysms with and without AWE.

	Non-AWE (*N* = 56)	AWE (*N* = 48)	Univariate *p*-Value	Multivariate *p*-Value	OR	95% CI
Site			0.85			
Left hemisphere	28 (50.00%)	23 (47.92%)				
Right hemisphere	28 (50.00%)	25 (52.08%)				
Height, median (IQR) (mm)	3.38 (2.21–4.51)	6.33 (4.40–8.05)	**<0.001**			
D_max_, median (IQR) (mm)	3.65 (2.74–4.69)	6.39 (4.85–8.89)	**<0.001**			
NW, median (IQR) (mm)	3.08 (2.44–4.00)	4.08 (3.26–5.39)	**0.003**			
Average diameter of parent arteries, median (IQR) (mm)	2.30 (2.12–2.50)	2.29 (2.05–2.60)	0.96			
AR, median (IQR)	1.01 (0.77–1.31)	1.62 (1.10–1.99)	**<0.001**	**0.001**	5.92	2.00–17.55
SR, median (IQR)	1.54 (0.99–2.07)	2.61 (2.06–3.45)	**<0.001**			
NSI, median (IQR)	0.11 (0.05–0.18)	0.20 (0.14–0.28)	**<0.001**			

NW, neck width; AR, aspect ratio; SR, size ratio; BNF, bottleneck factor; NSI, nonsphericity index; IQR, interquartile range; OR, odds ratio; CI, confidence interval.

**Table 3:** Comparison of the hemodynamics between high AR and low AR in patients with AWE.

	Low AR	High AR	*p*-Value
WSSen, median (IQR) (Pa)	0.6432 (0.4232–1.128)	0.0714 (0.0270–0.2036)	**<0.001**
WSSneck, median (IQR) (Pa)	3.458 (2.459–4.801)	2.987 (1.876–4.944)	0.48
Ven, median (IQR) (m/s)	0.0354 (0.0225–0.0476)	0.0083 (0.0012–0.0161)	**<0.001**
Vneck, median (IQR) (m/s)	0.1597 (0.1182–0.2706)	0.1448 (0.1055–0.2193)	0.45
WSSen/WSSneck, median (IQR)	0.1714 (0.1021–0.4702)	0.0227 (0.0077–0.0601)	**<0.001**
Ven/Vneck, median (IQR)	0.1806 (0.0930–0.3920)	0.0378 (0.0121–0.1086)	**<0.001**

WSSen, WSS at the enhanced area of aneurysm; WSSneck, WSS at the neck of the aneurysm; Ven, velocity beneath the enhanced area of aneurysm; Vneck, inflow velocity through the neck of the aneurysm; IQR, interquartile range.

**Table 4:** Comparison of the LVAR and CR_stalk_ between high AR and low AR in patients with AWE.

	Low AR	High AR	*p*-Value
LVAR, median (IQR)	0.1209 (0.0932–0.3188)	0.4669 (0.2266–0.5601)	0.007
CR_stalk_, median (IQR)	0.7338 (0.6406–0.8476)	0.8481 (0.7287–0.9969)	0.037

LVAR, ratio of low velocity area; IQR, interquartile range.

## Data Availability

The data presented in this study are available upon reasonable request from the corresponding author.

## Supplementary Materials

The following supporting information can be downloaded at: https://www.mdpi.com/article/10.3390/diagnostics14232722/s1, Figure S1: Definitions of aneurysm morphological parameters; Figure S2: Quantitative correlation of LVAR with the AR of the patients’ aneurysms; Table S1: CFD protocol used in the study.
