# Supplementary material for: Near-Wall Slow Flow Contributes to Wall Enhancement of Middle Cerebral Artery Bifurcation Aneurysms on Vessel Wall MRI
Source: Diagnostics (Basel). 2024 Dec 3;14(23):2722. doi: 10.3390/diagnostics14232722 (PMC11639947; doi:10.3390/diagnostics14232722)
Supplement: Supplementary file 1 [file diagnostics-14-02722-s001.zip › Table S1.pdf]

Table S1. CFD protocol used in the study.

	Patients' aneurysms	Silicone models
Time status	Transient	Steady
Inlet boundary condition	the pulsatile velocity profile of the MCA	0.1 m/s, 0.2 m/s, 0.3 m/s, 0.4 m/s, 0.5 m/s and 0.6 m/s
Outlet boundary condition	Zero static pressure	Zero static pressure
Time-step size (s)	0.02	-
Number of time-steps	100	-
