# Supplementary figures and images for: Near-Wall Slow Flow Contributes to Wall Enhancement of Middle Cerebral Artery Bifurcation Aneurysms on Vessel Wall MRI
Source: Diagnostics (Basel). 2024 Dec 3;14(23):2722. doi: 10.3390/diagnostics14232722 (PMC11639947; doi:10.3390/diagnostics14232722)

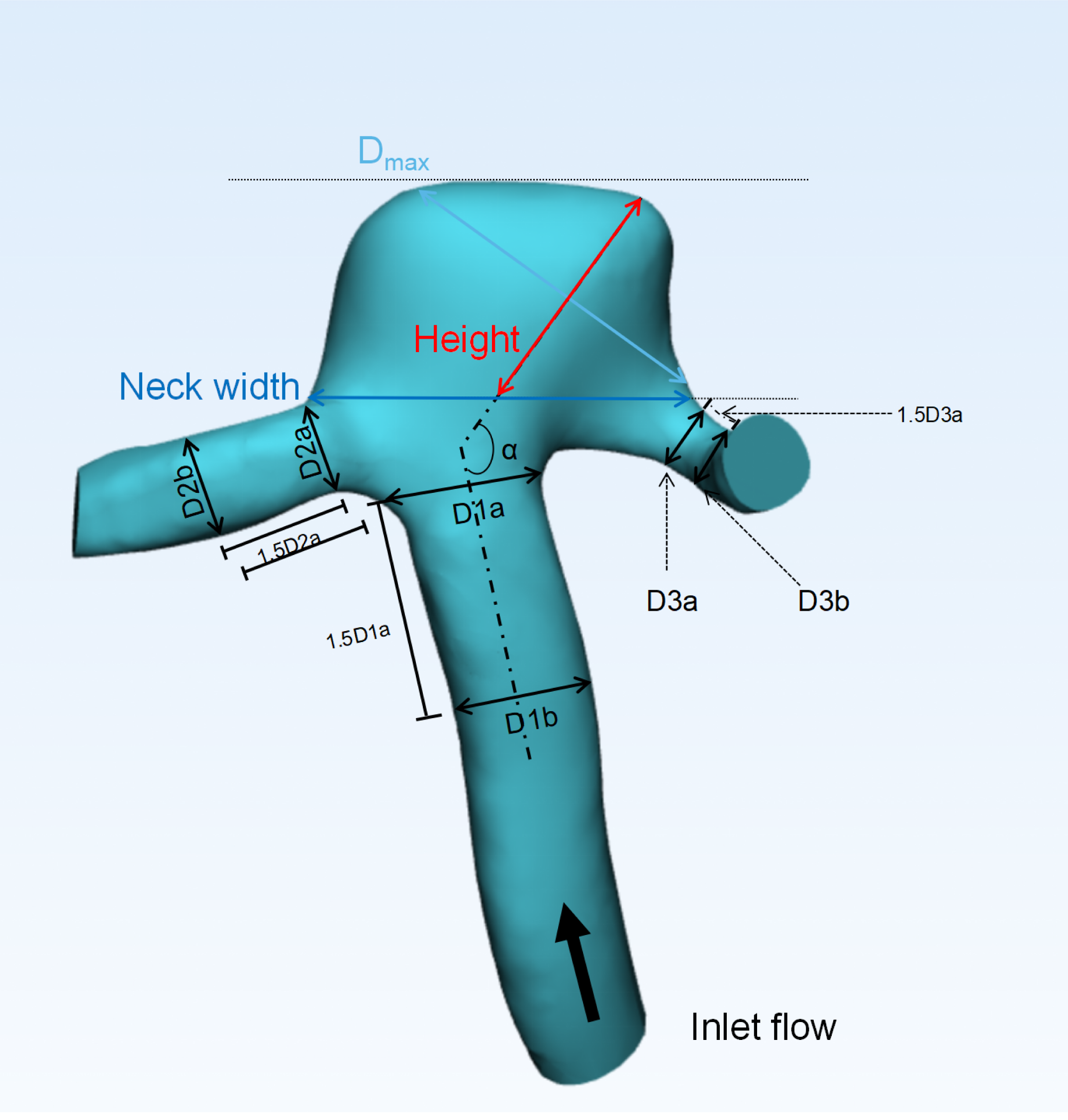

Supplement: Supplementary file 1 [file diagnostics-14-02722-s001.zip › Figure S1.tif]

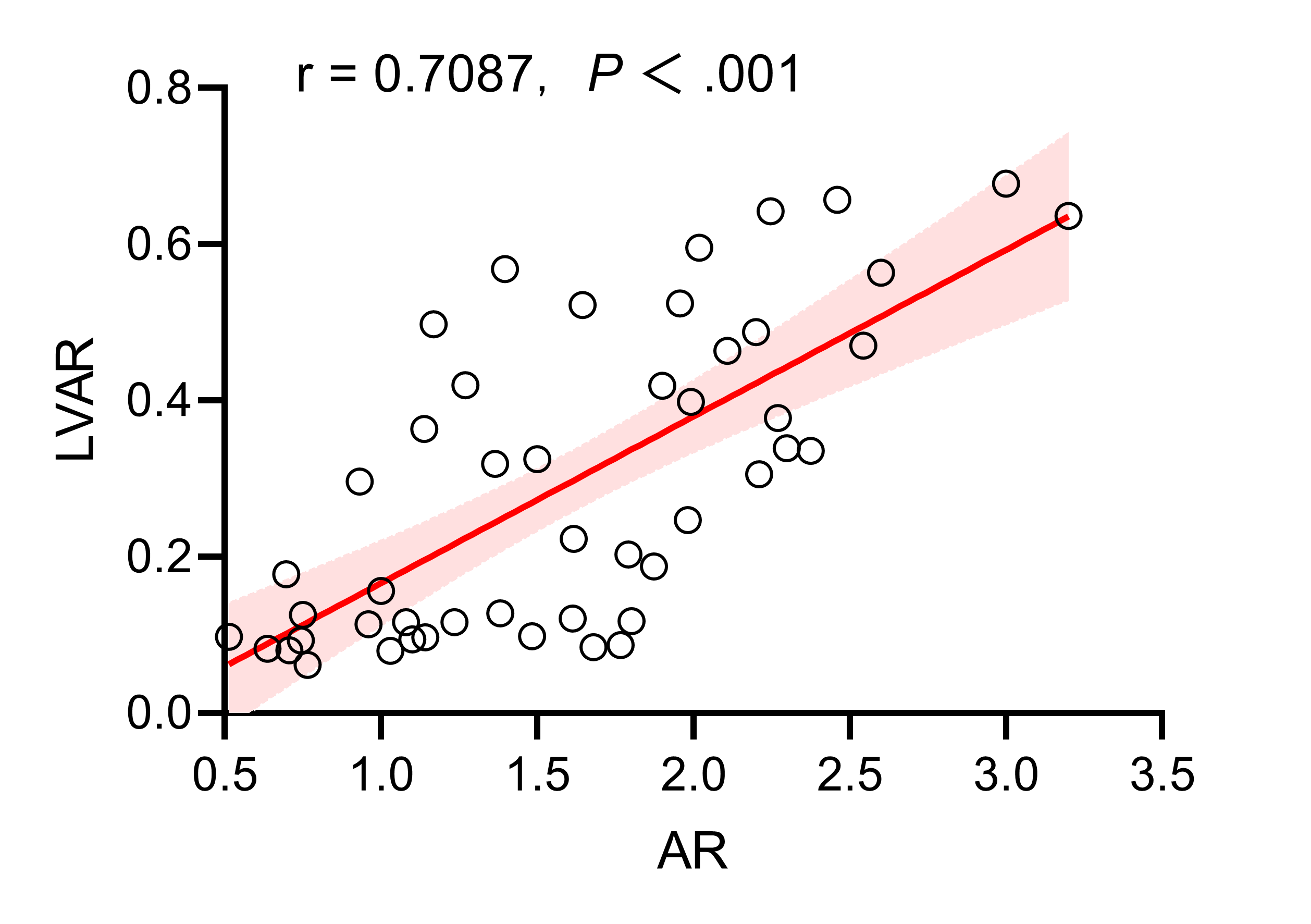

Supplement: Supplementary file 1 [file diagnostics-14-02722-s001.zip › Figure S2.tif]
